# The Kinematic and Electromyographic Analysis of Roller Skating at Different Speeds on a Treadmill: A Case Study

**DOI:** 10.3390/s24175738

**Published:** 2024-09-04

**Authors:** Giulia Bongiorno, Giulio Sisti, Francesca Dal Mas, Helena Biancuzzi, Tiwana Varrecchia, Giorgia Chini, Alberto Ranavolo, Barbara Pellegrini, Lorenzo Bortolan, Luca Miceli

**Affiliations:** 1 Physiotherapy Department, Friuli Riabilitazione, 33080 Roveredo in Piano, Italy; gbgiorno@gmail.com; 2 IRCCS C.R.O. National Cancer Institute of Aviano, 33081 Aviano, Italy; giulio.sisti@cro.it; 3 Department of Management, Ca’ Foscari University of Venice, 30121 Venice, Italy; francesca.dalmas@unive.it; 4 Collegium Medicum, University of Social Sciences, 90-229 Lodz, Poland; 5 Department of Economics, Ca’ Foscari University of Venice, 30121 Venice, Italy; helena.biancuzzi@unive.it; 6 Department of Occupational and Environmental Medicine, Epidemiology and Hygiene, INAIL, Monte Porzio Catone, 00078 Rome, Italy; g.chini@inail.it (G.C.); a.ranavolo@inail.it (A.R.); 7 CeRiSM, Sport Mountain and Health Research Center, University of Verona, 38068 Rovereto, Italy; barbara.pellegrini@univr.it (B.P.); lorenzo.bortolan@univr.it (L.B.); 8 Department of Engineering for Innovation Medicine, University of Verona, 37134 Verona, Italy; 9 Department of Pain Medicine, IRCCS C.R.O. National Cancer Institute of Aviano, 33081 Aviano, Italy; luca.miceli@cro.it

**Keywords:** surface electromyography, co-activation, skating, treadmill, elite athletes

## Abstract

Elite athletes in speed roller skates perceive skating to be a more demanding exercise for the groin when compared to other cyclic disciplines, increasing their risk of injury. The objective of this study was to monitor the kinematic and electromyographic parameters of roller speed skaters, linearly, on a treadmill, and to compare different skating speeds, one at 20 km/h and one at 32 km/h, at a 1° inclination. The acquisition was carried out by placing an inertial sensor at the level of the first sacral vertebra, and eight surface electromyographic probes on both lower limbs. The kinematic and electromyographic analysis on the treadmill showed that a higher speed requires more muscle activation, in terms of maximum and average values and co-activation, as it not only increases the intrinsic muscle demand in the district, but also the athlete’s ability to coordinate the skating technique. The present study allows us to indicate not only how individual muscle districts are activated during skating on a surface different from the road, but also how different speeds affect the overall district load distributions concerning effective force, which is essential for the physiotherapist and kinesiologist for preventive and conditional purposes, while also considering possible variations in the skating technique in linear advancement.

## 1. Introduction

With its parallels to field hockey and ice skating, speed roller skating is a cyclic sport in which the active and passive locomotor apparatus is subjected to significant stress. The groin region has been found to be the most vulnerable to the injuries seen in these sports in the scientific literature. For instance, it has been shown that in field hockey, about 10% of injuries affect the groin district in acute or chronic conditions [1,2]. Furthermore, within a sport season, about 20% of professional athletes have suffered an injury not induced by traumatic events, but by overload or incorrect technique during performance, with significant consequences on their seasonal performance [3,4].

Studies conducted on field hockey, and recently on speed roller skating, show that the most engaged muscles are the gluteus major, the gluteus medius, and the vastus lateralis, which are active during the propulsion phase, through maximal hip and knee extension, along with the same abduction and extra rotation [5]. Furthermore, the posterior thigh districts are most active during the maximum gliding phase, through isometric contraction, to stabilize the knee joint with the respective coactivation of the knee extensors [6]. Regarding skating, the activity of the leg districts implies a large activation of the anterior tibialis during both the final phases of propulsion and the recovery induced by the ankle being forced into dorsal flexion [7].

Analyzing skating performed on a treadmill may be useful for monitoring multiple muscle behaviors, which could help movement therapists in post-injury rehabilitation and athletic reconditioning. To date, the discipline that most observes the differences between road surfaces and treadmills, in terms of ground impact, joint loading, biomechanics, district acceleration, and the usefulness of the conveyor belt for rehabilitation and reconditioning purposes, purely concerns ground running activity [8,9].

To the best of our knowledge, there are no studies on the kinematics and sEMG analysis of skating on a treadmill, so the present study results in the first evaluative and comparative framework carried out in this context.

However, there is a marked difference here to the cyclic pattern of running, in that the propulsive action of skating focuses on a very pronounced abduction of the lower limb.

This movement is particularly evident at the hip, with propulsion being achieved by the extension, abduction, and external rotation of the entire lower district [10].

To better understand the muscle activation on both roads and treadmills, it has been seen that the greatest load at the adductor is induced by the purely eccentric cyclic action that forces the limb to decelerate during the propulsive phase of skating [11,12].

In this study, we aim to perform an electromyographic and kinematic analysis of a professional athlete on a treadmill at two speeds to understand changes in terms of muscle activations and coactivations and kinematics, which may, in the long run, affect the onset of overload issues. To do this, we also analyzed the propulsion and recovery phases of the lower limb districts and the body accelerations through the entire cycle. This would allow us to verify both the muscular and coordination requirements of the skating athlete. The programming of this discipline must, in fact, take into consideration how the values acquired in kinematics and muscle activation change during higher speeds. This is the basic concept on which these athletes’ preventive and conditional foundation is based, subjected not only to high speeds, but also to how these can influence their skating technique overall.

## 2. Materials and Methods

### 2.1. Participant

The participant is an elite female athlete (30 years old, body weight of 50 kg, and stature of 160 cm), and a former Italian, European, and world champion in speed roller skating. She is a right-handed person. She is in good health, with no musculotendinous, joint, or other clinical pathologies.

### 2.2. Electromyographic and Inertial Measurement Unit Recordings

Surface myoelectric signals were acquired (sampling rate of 1000 Hz) using a 16-channel Wi-Fi transmission surface electromyograph (FreeEMG300 System, BTS, Milan, Italy). Bipolar Ag/AgCl surface electrodes (24 mm diameter, H124SG Kendall ARBO, Tyco Healthcare, Neustadt/Donau, Germany) prepared with electroconductive gel were placed over each muscle [13]. Bipolar electrodes were placed bilaterally on the soleus (SOL), tibialis anterior (TA), rectus femoris (RF), biceps femoris (BF), gluteus maximus (GMax), gluteus medius (GMed), adductor longus (AL), and vastus lateralis (VL). In the region of electrode application, the skin was shaved, lightly scrubbed with sandpaper, and cleaned with alcohol.

A triaxial accelerometer (200 Hz, G sensor, BTS Bioengineering, Corp., Garbagnate Milanese, Italy) was positioned at the S1 level of the participant, medially, and fixed by means of the supplied adjustable strap.

### 2.3. Experimental Procedures

The participant performed the following specific exercises [14] that were needed to record the muscle activity during the isometric maximum voluntary contractions (iMVCs) for each of the muscles investigated:

(1) Push against a wall while standing on the sole of the foot;
(2) Dorsal flexion of the foot while sitting with manual resistance;
(3) Knee extension while sitting with fixed tibia;
(4) Knee flexion in a prone position with manual resistance;
(5) Hip flexion against manual resistance in a supine position;
(6) Hip extension in a prone position;
(7) Hip abduction against manual resistance in a supine position;
(8) Hip adduction in a semi-sitting position against a foam roller tented between the thighs.

The recorded iMVC signals were used to normalize the amplitude of the sEMG signals.

The participant was asked to perform inline skating with roller skates on a motorized treadmill with a belt surface 2.5 m wide and 3.5 m long (RL3500E, Rodby Innovation AB, Vänge, Sweden) at two different speeds, first at 20 km/h and then 32 km/h, with an inclination of 1°. The two trials at two speeds were performed half an hour apart to avoid confounding effects due to muscle fatigue.

### 2.4. Data Analysis

#### 2.4.1. Skating Cycle Definition

**Figure 1:**
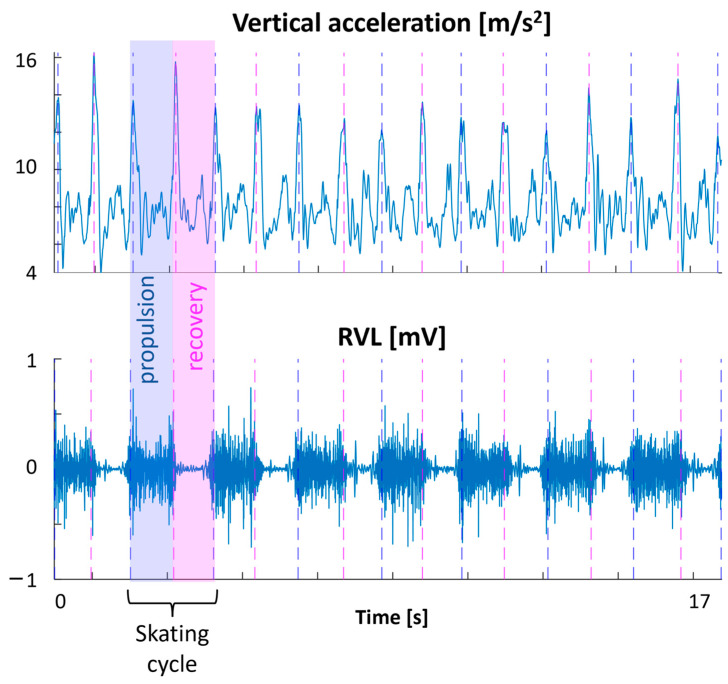
An example of skating cycles, propulsion, and recovery phase definitions, considering the vertical acceleration and the muscle data. RVL: right vastus lateralis.

The skating cycle was defined starting from the vertical acceleration ($a_{v}$) and the muscle data (Figure 1). The acceleration peaks were identified. Then, looking at the VL or GMed muscle [15], the propulsion phase was identified as the time in which the muscle was active (from the first to the second peak of $a_{v}$ within a skating cycle) and the recovery phase as the time in which the muscle was deactivated (from the second to the third peak of $a_{v}$ within a skating cycle). We identified nine skating cycles at 20 km/h and nine at 32 km/h. Then, to average the different cycles and compare the different speeds, we time-normalized all the skating cycle acceleration and EMG data, after the pre-processing described below, with a polynomial procedure for the same number of samples (201 samples) [16].

#### 2.4.2. sEMG

The sEMG signals were processed as follows: the iMVC and the sEMG raw data of each trial were band-pass filtered (4th-order Butterworth filter) between 20 and 400 Hz [17,18]; subsequently, a full-wave rectification of the signals was performed and low-pass filtering (4th-order Butterworth filter) at 10 Hz [19,20] was applied to extract the envelope of muscle activity; the rectified and filtered sEMG data related to each skating cycle were expressed as a percentage of the sEMG peak value [21,22,23], calculated as the maximum values detected for each of the iMVCs [21,22,24,25,26].

From the elaborated sEMG signals of each cycle, we computed the maximum value (Max) and the average rectified value (ARV) within the cycle.

Furthermore, we considered the following four muscles groups:

- RVL, RBF, RTA, and RSOL (group A);
- LVL, LBF, LTA, and LSOL (group B);
- RGMax, RRF, RGMed, and RAL (group C);
- LGMax, LRF, LGMed, and LRAL (group D).

For each group (A, B, C, and D), we calculated the simultaneous activation of the muscles (coactivation) by considering the Rudolph co-activation function for each pair of antagonist muscles [27] as follows:

(1) $$RC\left(k\right)=\left[{sEMG}_{H}\left(k\right)+{sEMG}_{L}\left(k\right)\right]\times \left[{sEMG}_{L}\left(k\right)/{sEMG}_{H}\left(k\right)\right]$$

where k is the k_th_ sample of the sEMG signals and $s{EMG}_{H}$ and ${sEMG}_{L}$ are the highest and the lowest activity between the antagonist muscle pairs.

Furthermore, we calculated the time-varying multi-muscle co-activation function (TMCf) proposed by Ranavolo and colleagues [28].

(2) $$TMCf\left(d\left(k\right),k\right)=\left(1-\frac{1}{1+e^{-12\left(d\left(k\right)-0.5\right)}}\right).\frac{{\;(\sum_{m=1}^{M}{sEMG}_{m}(k)/M)\;}^{2}}{{max}_{m=1\ldots M}[{sEMG}_{m}\left(k\right)]}$$

where d(k) is the mean of the differences between the k_th_ samples of each pair of sEMG signals:

(3) $$d(k)=\frac{\sum_{m=1}^{M-1}\sum_{n=m+1}^{M}\left|{sEMG}_{m}\left(k\right)-{sEMG}_{n}(k)\right|}{J(M!/(2!\left(M-2\right)!))}$$

In the above equations, J is 200 (the length of the signal); M is the number of considered muscles; and ${sEMG}_{m}\left(k\right)$ and ${sEMG}_{n}\left(k\right)$ are the k_th_ sample values of the envelope of the sEMG signals of the *m*th and *n*th muscles, respectively.

Then, from each co-activation function, we computed the Max and the ARV within the cycle.

## 3. Results

**Figure 2:**
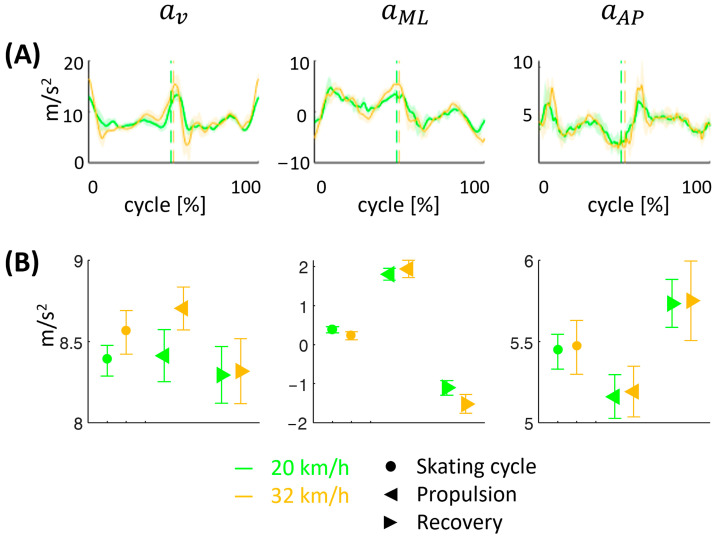
The mean vertical ($a_{v}$), medio-lateral ($a_{ML}$), and antero-posterior ($a_{AP}$) acceleration curves (**A**) during the skating cycles at two velocities (20 and 32 km/h) and the mean values (±SD) of the $a_{v}$, $a_{ML}$, and $a_{AP}$ accelerations (**B**) within the skating cycles and the propulsion and recovery phases at two velocities.

Figure 2A shows the mean vertical ($a_{v}$), medio-lateral ($a_{ML}$), and antero-posterior ($a_{AP}$) acceleration curves during the skating cycles at two velocities (20 and 32 km/h). The vertical lines represent the transition event (mean among all cycles) from the propulsion phase to the recovery phase. Figure 2B shows the mean values (±SD) of the $a_{v}$, $a_{ML}$, and $a_{AP}$ within the skating cycles and the propulsion and recovery phases at two velocities (20 and 32 km/h).

**Figure 3:**
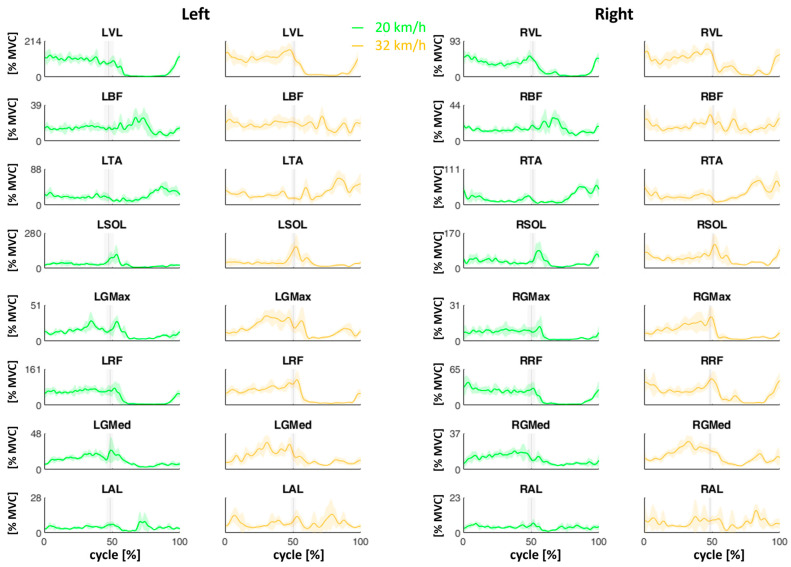
The mean (±SD) muscles curves during the skating cycles at two velocities (20 and 32 km/h). The vertical lines represent the mean (±SD) transition event from the propulsion phase to the recovery phase. SOL: soleus; GMax: gluteus maximus; GMed: gluteus medius; AL: adductor longus; RF: rectus femoris; BF: biceps femoris; VL: vastus lateralis; TA: tibialis anterior.

Figure 3 shows the mean (±SD) muscle curves during the skating cycles at two velocities (20 and 32 km/h). The vertical lines represent the mean (±SD) transition event from the propulsion phase to the recovery phase.

**Figure 4:**
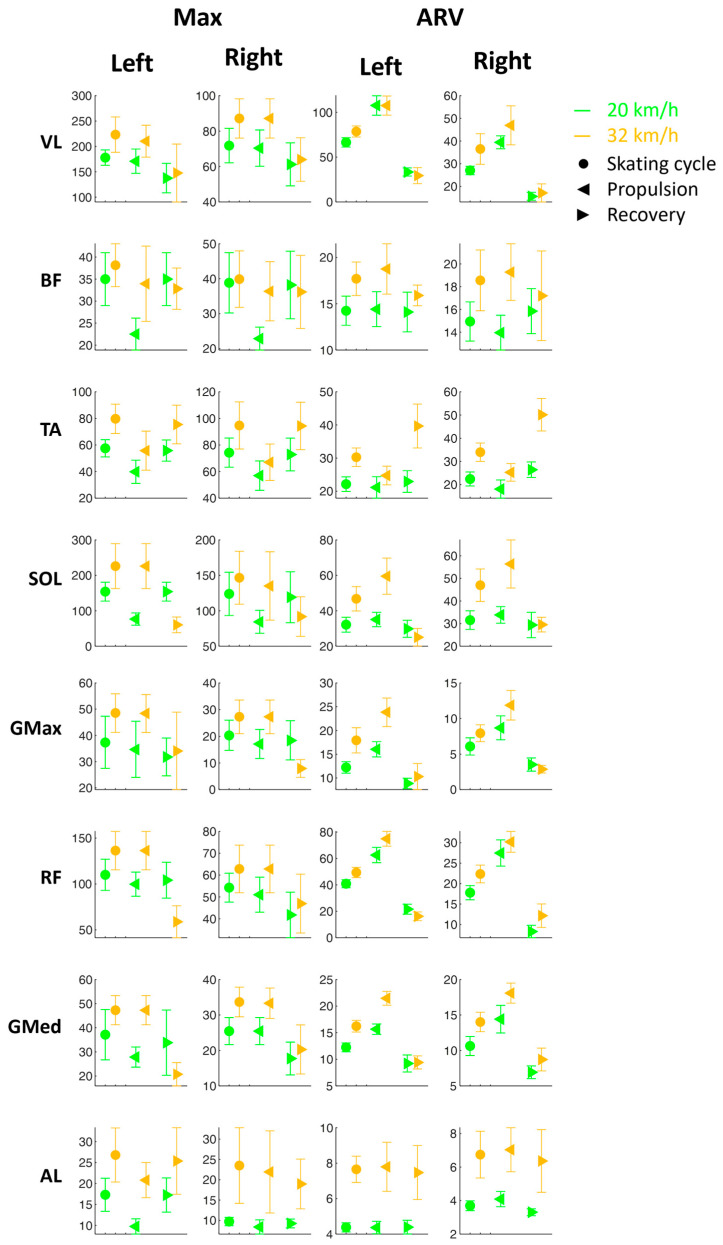
Maximum value (Max) and average rectified value (ARV) within the cycle. (±SD) for each muscle curve during the skating cycles and the propulsion and recovery phases at two velocities (20 and 32 km/h). SOL: soleus; GMax: gluteus maximus; GMed: gluteus medius; AL: adductor longus; RF: rectus femoris; BF: biceps femoris; VL: vastus lateralis; TA: tibialis anterior.

Figure 4 shows the Max and ARV (±SD) for each muscle curve during the skating cycles and the propulsion and recovery phases at two velocities (20 and 32 km/h).

**Figure 5:**
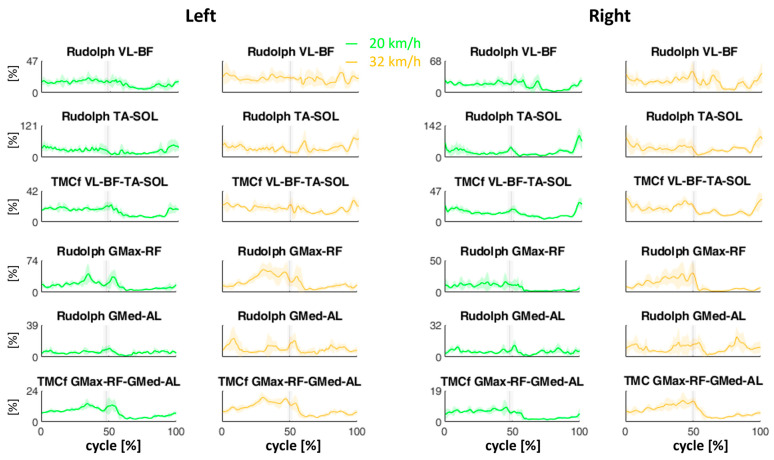
Mean (±SD) Rudolph and TMCf coactivation curves during the averaged skating cycle at two velocities (20 and 32 km/h). The vertical lines represent the mean (±SD) transition event from the propulsion phase to the recovery phase. SOL: soleus; GMax: gluteus maximus; GMed: gluteus medius; AL: adductor longus; RF: rectus femoris; BF: biceps femoris; VL: vastus lateralis; TA: tibialis anterior.

Figure 5 shows the mean (±SD) Rudolph coactivation curves for each pair of muscles and the TMCf coactivation curves for each muscle group, A, B, C and D, during the skating cycles at two velocities (20 and 32 km/h). The vertical lines represent the mean (±SD) transition event from the propulsion phase to the recovery phase.

**Figure 6:**
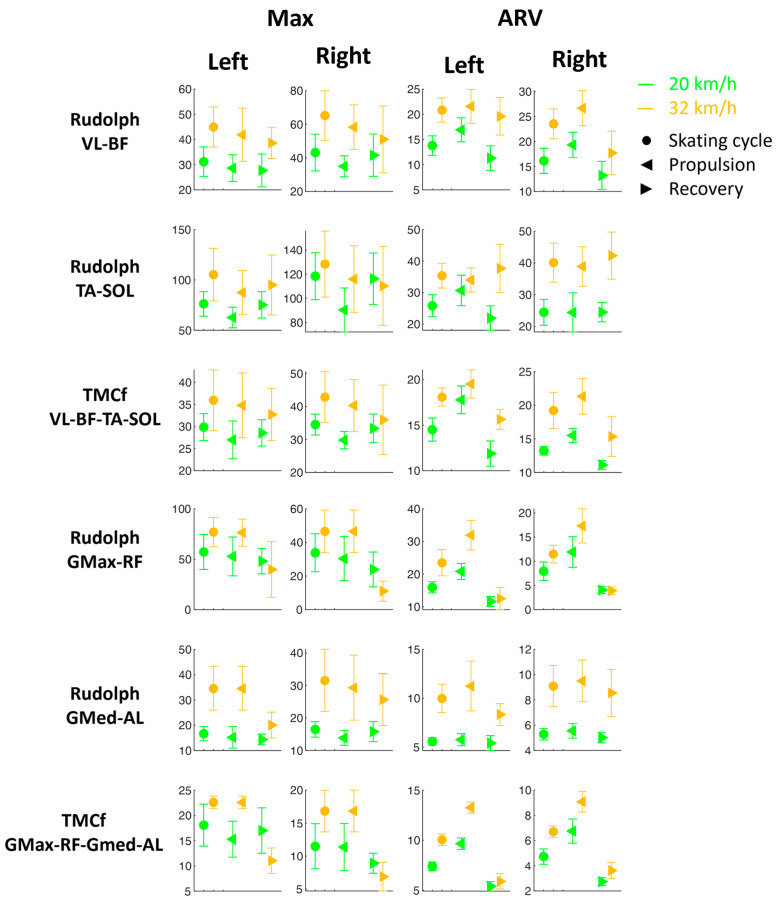
Maximum value (Max) and average rectified value (ARV) within the cycle (±SD) for each muscle curve during the skating cycles and the propulsion and recovery phases at two velocities (20 and 32 km/h). SOL: soleus; GMax: gluteus maximus; GMed: gluteus medius; AL: adductor longus; RF: rectus femoris; BF: biceps femoris; VL: vastus lateralis; TA: tibialis anterior.

Figure 6 shows the Max and ARV (±SD) for each Rudolph and TMCf coactivation curve during the skating cycles and the propulsion and recovery phases at two velocities (20 and 32 km/h).

## 4. Discussion

With this case study, we aimed to investigate the spatial–temporal and muscular behavior of a professional female athlete on a two-speed treadmill.

From a kinematics point of view, the average value of the vertical component of acceleration is increased at higher speeds throughout the entire skating cycle and in the propulsion phase (Figure 2b). This is mainly due to the need, since the athlete is stationary along the antero-posterior direction, to gain speed in the push phase. The increased vertical component of the acceleration is also associated with an increase in the duration of the propulsion phase. This speed-based induced motor mechanism suggests the possibility of training the technical gesture and the reference muscles in targeted re-athleticization or athletic preparation programs.

From an analysis of the results of the muscular behaviors, it can be seen that higher velocities imply higher muscle commitments (Figure 4). In fact, the VL, BF, TA, SOL, GMax, RF, GMed, and AL maximum values increased at 32 km/h in the entire skating cycle and in the propulsion phase. The increase in the peak activation of the hip, knee, and ankle extensor muscles explains the increase in the vertical component of the acceleration.

In terms of the ARV, however, it is possible to observe a significant increase with speed in the VL, BF, SOL, GMed, and AL muscles in the skating cycle and the propulsion phase (Figure 4). This is attributable to the need to manage a wider propulsion phase and a greater push along the vertical component for the entire duration of the propulsion phase itself.

For the TA and AL, then, in terms of both Max and ARV, there is also an increase with speed in the recovery phase, as shown in Figure 4. This is due to the need to recover an adequate posture for the subsequent propulsion phase.

In general, both the coactivation calculated with Rudolph’s approach and TMCf show highest values at 32 km/h, both in terms of the Max and ARV in the skating cycle and in both the propulsion and recovery subphases (Figure 6). This is in line with what has also been reported in other studies on locomotor tasks such as walking and running [29,30,31].

These results suggest that the contribution to = limb stiffening during skating is mostly due to the muscle co-activation of the extensors according to their function in load acceptance and their propulsive role [32]. This of also interest because the flexor muscles play a key role in the transition from the propulsive to the recovery phase.

The only exception is found in the Max of the Rudolph coactivation for the GMax-RF pair and for the Max of the TMCf for the GMax-RF-GMed-AL, which shows a slight reduction in the recovery phase at the highest speed (Figure 6).

In addition, from Figure 4, a consistent symmetry between the left and right sides can be observed in general, excluding VL, with RF showing much higher values on the left side than on the right side; TA, SOL, GMax, and GMed show slightly higher values on the left than on the right, both in the full cycle and in the propulsion and recovery phases. This asymmetry could be due to the fact that the athlete is right-handed and needs to activate the muscle of the non-dominant side more. From Figure 6, on the other hand, greater symmetry emerges between the left and right sides in the coactivation parameters (Max and ARV), both in the whole skating cycle and in each of the two subphases. These results confirm the symmetrical nature of the motor task on the treadmill, unlike what happens on the road where, in curvilinear sections, there can be an asymmetrization between the two sides [33]. In skating, training should be designed to minimize the risk of muscle fatigue asymmetry and to decrease asymmetry [34]. In this regard, the literature has provided a functional protocol of analysis on roads, which, through surface electromyographic instrumentation, allows for the monitoring of both kinematics and muscle activation of the investigated districts in a professional athlete. This study incorporates the same acquisition methods and instrumentation already used on the skating performance of a former world champion athlete, serving as a performance model [15,35].

The results of this study must be considered absolutely preliminary, as they can be attributed to a case study. On the other hand, the differences induced by the two speeds set on the treadmill suggest the need to investigate a larger sample in order to analyze any differences in gender (we have analyzed only a female), age, right- and left-handed people, and the experience of the athlete.

## 5. Conclusions

Performing the skating task on a treadmill at different speeds can be particularly useful for inducing different motor behaviors, with the aim of training different functions. Furthermore, the treadmill allows for the careful evaluation of the technical gestures, also due to the greater simplicity in carrying out instrumental measurements with wearable sensors.

## Data Availability

The dataset is available on request from the authors.
